# Impact of Changes in Infection Control Measures on the Dynamics of COVID-19 Infections in Schools and Pre-schools

**DOI:** 10.3389/fpubh.2021.780039

**Published:** 2021-12-20

**Authors:** Martina Sombetzki, Petra Lücker, Manja Ehmke, Sabrina Bock, Martina Littmann, Emil C. Reisinger, Wolfgang Hoffmann, Anika Kästner

**Affiliations:** ^1^Department of Tropical Medicine and Infectious Diseases, University Medical Center Rostock, Rostock, Germany; ^2^Department for Epidemiology of Health Care and Community Health, Institute for Community Medicine, University Medicine Greifswald, Greifswald, Germany; ^3^Landesamt für Gesundheit und Soziales Mecklenburg-Vorpommern State Office for Health and Social Affairs, Rostock, Germany

**Keywords:** schools and pre-schools, routine surveillance data, control measures, multivariate regression analysis, SARS-CoV-2 infections

## Abstract

**Introduction:** With the increased emergence of SARS-CoV-2 variants, the impact on schools and preschools remains a matter of debate. To ensure that schools and preschools are kept open safely, the identification of factors influencing the extent of outbreaks is of importance.

**Aim:** To monitor dynamics of COVID-19 infections in schools and preschools and identify factors influencing the extent of outbreaks.

**Methods:** In this prospective observational study we analyzed routine surveillance data of Mecklenburg-Western Pomerania, Germany, from calendar week (CW) 32, 2020 to CW19, 2021 regarding SARS-CoV-2 infection events in schools and preschools considering changes in infection control measures over time. A multivariate linear regression model was fitted to evaluate factors influencing the number of students, teachers and staff tested positive following index cases in schools and preschools. Due to an existing multicollinearity in the common multivariate regression model between the variables “face mask obligation for children” and “face mask obligation for adults”, two further separate regression models were set up (Multivariate Model Adults and Multivariate Model Children).

**Results:** We observed a significant increase in secondary cases in preschools in the first quarter of 2021 (CW8 to CW15, 2021), and simultaneously a decrease in secondary cases in schools. In multivariate regression analysis, the strongest predictor of the extent of the outbreaks was the teacher/ caregiver mask obligation (*B* = −1.9; 95% CI: −2.9 to −1.0; *p* < 0.001). Furthermore, adult index cases (adult only or child+adult combinations) increased the likelihood of secondary cases (*B* = 1.3; 95% CI: 0.9 to 1.8; *p* < 0.001). The face mask obligation for children also showed a significant reduction in the number of secondary cases (*B* = −0.6; 95% CI: −0.9 to −0.2; *p* = 0.004.

**Conclusion:** The present study indicates that outbreak events at schools and preschools are effectively contained by an obligation for adults and children to wear face masks.

## Introduction

Since the emergence of the coronavirus SARS-CoV-2 and the subsequent global COVID-19 pandemic, the role of children and adolescents, and in particular the role of schools and pre-schools in the infection process remains unclear. Preliminary results suggest that children younger than 10–14 years have a lower susceptibility to SARS-CoV-2 infection compared to adults (1–4). In addition, children have lower rates of severe COVID-19 courses compared to other age groups and present fewer and milder symptoms compared to adults (5–7).

Although the closure of general and private schools, as well as pre-schools, has become a common approach in containing the COVID-19 pandemic, the contribution of school and pre-school openings to the dynamics of the pandemic is unclear. On the one hand, it has been shown that schools play a rather minor role in virus spreading and that infections occurring in schools largely reflect the incidence of the surrounding area (8, 9). On the other hand, it has been shown that school closures can have a significant effect on the trend reversal of case numbers (10, 11). Nevertheless, early modeling studies of COVID-19 suggested that school closures alone would prevent only 2–4% of overall COVID-19-associated deaths (12). Children and adolescents represent a vulnerable group that is particularly at risk due to the social deprivation and constraints imposed by school closures, with consequent negative physical, psychological, and educational effects (13, 14).

To date, there is only insufficient evidence to definitively rule out schools as a source of infection. It remains undisputed that children and adolescents can be infected with SARS-CoV-2 and can spread the infection possibly as asymptomatic carriers. However, studies in schools suggest that infections are predominantly brought into schools by adults and that a child-to-child transmission in schools is rare and probably not the primary cause of SARS-CoV-2 infections in children collectives (15, 16). An analysis of laboratory-confirmed COVID-19 cases in children and adolescents in Germany from January to August 2020 showed that affected schools had few cases per outbreak and that older age groups were affected more frequently (17). Actual COVID-19 outbreaks in schools, i.e., infection events with more than one person infected are rare (8, 17).

Because new virus variants with altered infection dynamics emerge, it is crucial to closely monitor infection events in schools and pre-schools. In a study by Loenenbach et al. children and adolescents showed a comparable secondary attack rate (SAR) upon infections with SARS-CoV-2 variant B.1.1.7 to adults, with consequent evidence for increased susceptibility and infectiousness of the viral mutant in children and adolescents (18). In addition, the delta variant of SARS-CoV-2 is spreading rapidly worldwide. Analyses of self-administered RT-PCR swab samples tested for SARS-CoV-2 positivity as well as viral genome sequencing suggest that children may be infected with the delta variant of the virus more frequently than adults (19, 20). In the absence of pre-existing conditions, however, there is currently no evidence of a higher risk of severe disease progression in this age group. Nevertheless, there is some evidence suggesting that a relevant proportion of children may experience long-term effects similar to adults after clinical COVID-19 infection (Long COVID) (21).

The aim of this prospective observational study was to monitor the dynamics of infection events at schools and pre-schools, taking into account the hygiene regulations in force in Mecklenburg-Western Pomerania at the time, and to identify factors influencing the extent of outbreaks.

## Methods

The following evaluations are based on data from the routine surveillance of the State Office for Health and Social Affairs Mecklenburg-Western Pomerania (Landesamtes für Gesundheit und Soziales M-V, LAGuS). We analyzed information of school-related infections from calendar week (CW) 32 in 2020 to CW 19 in 2021 at general and private schools and pre-schools. Local health offices identified school-related cases through contact tracing and reported them to the LAGuS. All vocational schools were excluded due to the special regulations with regard to hygiene measures and heterogeneity compared to the other types of schools. The primary objective of the study was to investigate the dynamics of COVID-19 infections in schools and pre-schools in dependence on pandemic-related changes in hygiene measures and to investigate the influence of hygiene measures on the extent of outbreaks. The ethics committee at the University Medical Center Rostock gave a positive vote on this study (Registration Number: A2020-0090).

### Study Population

Cases with SARS-CoV-2 positive results were identified by laboratory-based RT-PCR testing. Then, the positive test results were forwarded to the LAGuS by physicians or laboratory staff. Federal health authorities categorized the infections as index or secondary cases by identifying contact persons and the onset of symptoms. Furthermore, infected individuals were indicated as children or adults. The number of infections that occurred at each school/pre-school were listed according to the respective CW.

Information on the affected institution for this study were provided by the LAGuS. These included name and location, the number of infections among adults and children, their classification into index and secondary cases as well as the prescribed measures. The information was anonymized and did not convey personal data. In a few cases, it was either not possible to identify the first person infected, or infection events occurred simultaneously, so two individuals may be listed as the index case.

An infection event in schools/pre-schools was defined by the LAGuS as one person tested positive by PCR testing (index case) and, in temporal (maximum 10 days) and spatial relation (same institution) to this, the occurrence of another person tested positive by PCR testing (secondary case). The R-factor (R-F) was calculated by dividing the number of secondary cases by the number of index cases.

### Infection Control Measures and Timeline

Hygiene measures applicable in Mecklenburg-Western Pomerania were listed on the basis of the information letters and corona ordinances issued by the Ministry of Education, Science and Culture and the Ministry of Social Affairs, Integration and Equality in collaboration with the LAGuS (22, 23). The applicable hygiene measures were documented chronologically by date for schools and pre-schools (Table 1).

**Table 1:** Applicable hygiene measures in schools (Ministry of Education, Science and Culture) and pre-schools (Ministry of Social Affairs, Integration and Equality) in Mecklenburg-Western Pomerania comparing phase 1 with phase 3.

	**Phase 1 (CW32–CW51, 2020)**		**Phase 3 (CW8–CW15, 2021)**	
	**School**	**Pre-school**	**School**	**Pre-school**
Face mask obligatory in hallways, restrooms, schoolyard for staff and students	Yes, if not in one cohort or students grade 1–4	No	Yes	No
Face mask obligatory during lessons/in pre-schools for staff	No, until 16th of December 20	No, only in case of contact with a symptomatic child	Yes	No, only in case of adult-adult contact or contact with a symptomatic child
Face mask obligatory during lessons/in pre-schools for children	No	No	Yes	No
Medical face mask recommended	No	No	Yes	Yes
1.5-m minimum distance, if possible	Yes	Yes	Yes	Yes
1.5-m minimum distance during lessons	No	-	Yes	-
Grouping children into defined groups (cohorting)	Yes	Yes	Yes	Yes
Voluntary preventive PCR testing for employees	Yes	Yes	Yes	Yes
Voluntary preventive PoC testing for children	No	No	Yes, since 1st of March 21	No
Voluntary preventive PoC testing for staff	No	No	Yes, since 1st of March 21	Yes, since 1st of April 21
Mandatory PoC testing for staff	No	No	No	No
Mandatory PCR testing for children in case of symptoms	No	No	Yes, since 12th of April 21	Yes, since 12th of April 21
Vaccination Prioritization for staff	No	No	Yes	Yes

*PoC, Point-of-care SARS-CoV-2 antigen tests*.

To capture the impact of hygiene measures, we divided infection events throughout the school year 2020/2021 into four phases. In phase 1, both schools and pre-schools were open on a regular basis and in usual operation, subject to conditions such as maintaining a minimum distance of 1.5 m, wherever possible. In the second phase, schools and pre-schools were closed. In the third phase, schools and pre-schools were gradually opened with modified hygiene measures, depending on the local incidence levels (see Table 1). In the fourth phase, there was another lockdown. During the lockdown, schools and pre-schools were primarily closed. Pupils were instructed from the distance to self-study at home. Emergency care was provided for children from pre-school until 6th grade, if parents could prove to have system-relevant jobs (such as hospital employees, etc.) and were not able to organize other forms of childcare. Furthermore, all graduating classes were allowed to be taught in presence in the school building.

The study period was divided into the following time phases depending on the infection control measures in place:

**Table:** 

Phase 1:	CW32/20–CW51/20	School/pre-school opening phase
Phase 2:	CW52/20–CW7/21	2nd Lockdown Germany
Phase 3:	CW8/21–CW15/21	School/pre-school reopening phase
Phase 4:	CW16/21–CW19/21	3rd Lockdown Germany

### Statistical Analysis

Continuous variables were reported as median (range), and categorical data as counts and percentages. In addition, the mean value per infection event was reported for the following variables: overall cases, index cases, secondary cases, index cases children, index cases adults, secondary cases children and secondary cases adults. Univariate analysis for categorical variables was performed by using Chi^2^-test or Fisher's exact-test and for continuous variables with Student's *t*-Test or Mann-Whitney-*U* test depending on the normal distribution (tested with Shapiro-Wilk test). To evaluate the impact of associated variables on the number of secondary cases, a multivariate analysis was conducted.

For multivariate analysis a common linear regression model was set up with the number of secondary cases as the dependent variable and as independent variables the total number of index cases, type of index case [Child (1)/Adult (2)/Child + Adult (3)], Mask obligation for adults [No (0)/Yes, conditionally (1)/Yes, everywhere in school building/pre-school (2)], mask obligation for children {No (0)/[No/Yes, conditionally (1)]/Yes, conditionally (2)/Yes, everywhere in the school building/pre-school (3)} and setting [school (0)/pre-school (1)]. For the variable “affected person as index case,” it occurred that more than one person was documented as an index case. This means that it was not possible to distinguish which person was infected first, e.g., because symptoms occurred simultaneously or two outbreaks occurred at the same time. When a child and an adult were documented as index cases, they were assigned to the “child + adult” category. With respect to the variables “face mask mandatory for adults” and “face mask mandatory for children,” “Yes, under certain circumstances” was selected if the mask obligation existed only under certain circumstances but not during the entire time spent in the school building/pre-school (Table 1). For schools, for example, this applies if there was no mask obligation during lessons and in pre-schools if there was no mask obligation for adults during pedagogical work with the children. Furthermore, the “No/Yes, conditionally” category was applied when the school consisted of a primary and secondary part, due to different applicable hygiene measures for children among different age groups. Time and location were considered by CW and county of the school/pre-school and were dummy-coded in the model to avoid temporal and spatial associations. Due to multicollinearity between the variables mask obligation for children and mask obligation for adults, two further multivariate linear regression models were set up, whereby in the “Multivariate Model Adults” the mask obligation for children was not taken into account and in the “Multivariate Model Children” the mask obligation for adults was not considered. Regression coefficients (*B*) are presented with 95% confidence intervals (CI). The goodness of fit of the model was determined using *R*^2^ and the corrected *R*^2^. Cohen's *f*^2^ was calculated with the formula *f*^2^ = [corrected *R*^2^/(1-corrected *R*^2^)].

A *p*-value < 0.05 was considered statistically significant. Statistical analysis was performed using IBM SPSS Statistics version 27. The figures were created using Microsoft® Excel (Microsoft® Excel for Mac, Version 16.51 (21071101).

## Results

### Hygiene Measures in Schools and Pre-schools

The specifications for hygiene measures to contain the COVID-19 pandemic in schools and pre-schools were continually adapted and changed throughout the study period from August 2020 until May 2021. Since schools and pre-schools were open in phase 1 and phase 3, we particularly compared the hygiene measures in those periods. Table 1 provides an overview of the hygiene measures that were in place in schools and pre-schools during each phase. General hygiene measures such as keeping a minimum distance (at least 1.5 m), proper coughing, sneezing and thorough hand washing, wearing a mask and frequent ventilation were persistent throughout the study period and are therefore neglected in the overview.

Regulations differed between schools and pre-schools in Mecklenburg-Western Pomerania, particularly regarding the obligation to wear a mask covering mouth and nose. A differentiation between staff (educators and teachers) and children is relevant. Face masks became mandatory on school grounds and within school buildings at the 4th of August 2020. Defined exceptions applied, e.g., during lessons; for pupils from 1st til 4th grade; if a distance of at least 1.5-m was kept or while drinking and eating.

The mask obligation during lessons was introduced for teachers at schools on 16th of December 2020, whereas this obligation for children was issued 8th of January 2021. Children in pre-schools were not required to wear a mask at any time of our study period. Educators in pre-schools are exempt from the mask requirement during their pedagogical work with children.

Other hygiene measures, such as maintaining the 1.5 m minimum distance, building defined cohorts, and offering vaccination to staff members, did not differ between the settings in the respective phases.

### Infection Events in Schools

**Figure 1:**
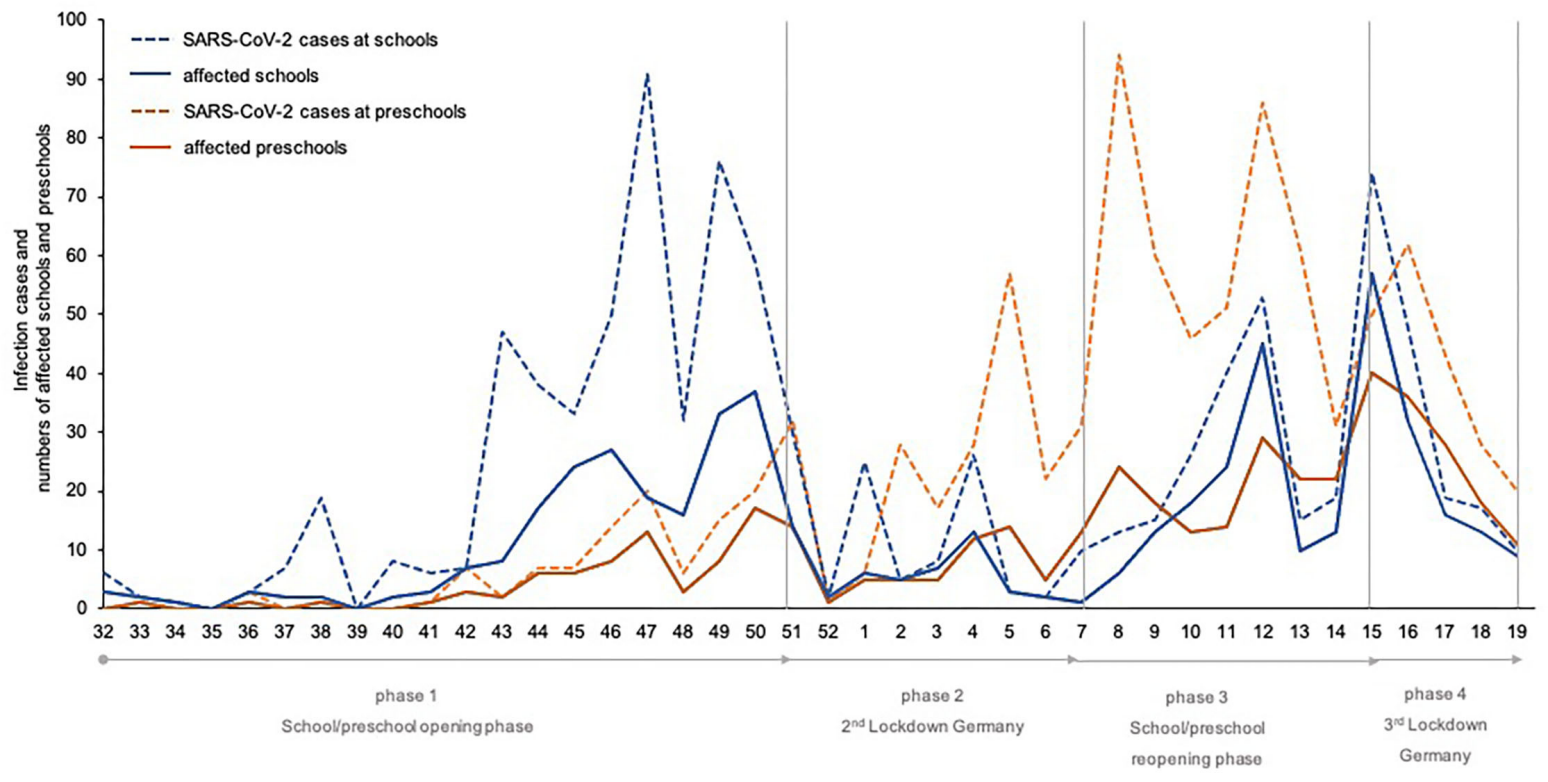
Total number of SARS-CoV-2 cases at schools and pre-schools in the study period (week 32/20–19/21) divided into the different phases of infection control measures in Mecklenburg-Western Pomerania.

A total of 956 infection events occurred during the study period, of which *n* = 43 infection events were excluded from the analyses because they occurred at vocational schools. Of the included *n* = 913 infections, *n* = 475 occurred in schools and *n* = 438 in pre-schools. A summary of the total number of SARS-CoV-2 cases at schools and pre-schools during the study period is provided in Figure 1.

In phases 1 (CW32-CW51, 2020) and 3 (CW8–CW15, 2021), the schools were open under different restrictions (Table 2). In phase 1, a total of *n* = 189 schools were affected by at least one infection. A total of *n* = 225 index cases and *n* = 236 secondary cases were identified (R-F = 1.05). Most frequently regional schools (*n* = 67) were affected, followed by elementary schools (*n* = 41) and integrated/cooperative, comprehensive schools (*n* = 35), grammar schools (*n* = 30), or special schools (*n* = 15).

**Table 2:** Infection incidence in schools in Mecklenburg-Western Pomerania subdivided by time phases depending on infection control measures in place, N = 475.

	**Phase 1** **(CW32-CW51, 2020)** ***n* = 189**	**Phase 2** **(CW52, 2020 – CW7, 2021)** ***n* = 37**	**Phase 3** **(CW8–CW15, 2021)** ***n* = 179**	**Phase 4** **(CW16-CW19, 2021)** ***n* = 70**	***p*-value** **Phase 1 vs.** **Phase 3**
Schools open	Yes	No	Yes	No	
Public school	169 (89.9)	28 (77.8)	160 (89.4)	57 (86.4)	0.873
Private school	19 (10.1)	8 (22.2)	19 (10.6)	9 (13.6)	
**County, no. (%)**					
Hansestadt Rostock	18 (9.5)	4 (10.8)	23 (12.8)	13 (18.6)	**0.003**
Landkreis Rostock	24 (12.7)	6 (16.2)	32 (17.9)	9 (12.9)	
Ludwigslust-Parchim	39 (20.6)	3 (8.1)	18 (10.1)	14 (20.0)	
Mecklenburgische Seenplatte	3 (1.6)	3 (8.1)	14 (7.8)	4 (5.7)	
Nordwest-Mecklenburg	31 (16.4)	4 (10.8)	25 (14.0)	6 (8.6)	
Schwerin	16 (8.5)	0 (0)	10 (5.6)	4 (5.7)	
Vorpommern-Greifswald	23 (12.2)	11 (29.7)	32 (17.9)	14 (20.0)	
Vorpommern-Rügen	35 (18.5)	6 (16.2)	25 (14.0)	6 (8.6)	
**School type, no. (%)**					**0.007**
Elementary school	41 (21.8)	13 (36.1)	70 (39.3)	27 (40.9)	
Special school	15 (8.0)	3 (8.3)	14 (7.9)	6 (9.1)	
Integrated/cooperative comprehensive school	35 (18.6)	5 (13.9)	24 (13.5)	9 (13.6)	
Regional school	67 (35.6)	11 (30.6)	46 (25.8)	22 (33.3)	
Grammar school	30 (16.0)	4 (11.1)	24 (13.5)	2 (3.0)	
**Average cases per infection event**					
Cases overall	2.44	1.86	1.57	1.34	0.084
Index cases	1.19	1.05	1.18	1.11	0.865
Secondary cases	1.25	0.81	0.39	0.23	**0.046**
Index cases children	0.93	0.59	0.96	0.97	0.412
Index cases adults	0.26	0.46	0.22	0.14	0.330
Secondary cases children	1.07	0.24	0.32	0.17	0.080
Secondary cases adults	0.17	0.57	0.07	0.06	0.103
Index cases overall, absolute no.	225	39	211	78	
Secondary cases overall, absolute no.	236	30	70	16	
R-Factor (R-F^*^)	1.05	0.77	0.33	0.21	
**Overall cases per infection event, no. (%)**					
One case overall	126 (66.7)	31 (83.8)	133 (74.3)	52 (74.3)	0.086
2–10 cases overall	57 (30.2)	6 (16.2)	45 (25.1)	18 (25.7)	
>10 cases overall	6 (3.2)	0 (0)	1 (0.6)	0 (0)	
**Affected person as index case, no. (%)**					
Child only	143 (75.7)	21 (56.8)	143 (79.9)	61 (87.1)	0.620
Adult only	38 (20.1)	16 (43.2)	30 (16.8)	7 (10.0)	
Child + adult^**^	8 (4.2)	0 (0)	6 (3.4)	2 (2.9)	
Index case child only, no. (R-F^*^)	160	22	161	65	
Secondary cases overall	42 (0.26)	10 (0.45)	53 (0.33)	10 (0.15)	0.841
Secondary cases adults	11 (0.07)	6 (0.27)	9 (0.06)	2 (0.03)	0.629
Secondary cases children	31 (0.19)	4 (0.18)	44 (0.27)	8 (0.12)	0.395
Index case adult only, no. (R-F^*^)	40	17	32	8	
Secondary cases overall	179 (4.48)	20 (1.18)	15 (0.47)	5 (0.63)	**0.002**
Secondary cases adults	22 (0.55)	15 (0.88)	4 (0.13)	2 (0.25)	0.059
Secondary cases children	157 (3.93)	5 (0.29)	11 (0.34)	3 (0.38)	**0.001**
**Index case child** **+** **adult, no**.	25	0	18	5	
(child | adult)	(15 | 10)	(0 | 0)	(11 | 7)	(3 | 2)	
Secondary cases overall	15	0	2	1	
(child | adult)	(15 | 0)	(0 | 0)	(2 | 0)	(1 | 0)	
**Measure for containment, no. (%)**					**<0.001**
Contact person quarantine	41 (22.3)	17 (48.6)	83 (46.9)	38 (55.9)	
Cohort quarantine	128 (69.6)	16 (45.7)	90 (50.8)	30 (44.1)	
Closure of the entire facility	15 (8.2)	2 (5.7)	4 (2.3)	0 (0)	

* *R-F, R-Factor*.

** *This category was applied when a child and an adult were documented as index cases because it could not be differentiated which person was infected first, for example, because symptoms may have occurred at the same time or two outbreaks may have occurred simultaneously*.

*Values in bold are considered to be statistically significant (p-value < 0.05)*.

In phase 3 (CW8-CW15 2021, open schools), a comparable number of schools was affected (*n* = 179) as in phase 1, but the incidence shifted to younger school cohorts (elementary schools *n* = 70, regional Schools *n* = 46, grammar schools *n* = 24, integrated/cooperative, comprehensive schools *n* = 24, special schools *n* = 14. A total of *n* = 211 index and *n* = 70 secondary cases (R-F = 0.33) were identified in phase 3.

In both phases 1 and 3, children were identified more frequently as index cases (phase 1: *n* = 160 and phase 3: *n* = 161) compared to adults (phase 1: *n* = 40 and phase 3: *n* = 32). The number of secondary cases caused by the index case “child only” was also comparable in both phases (phase 1: R-F = 0.26 and phase 3: R-F = 0.33), whereby children became infected more frequently (phase 1: R-F = 0.19 and phase 3: R-F = 0.27) than adults (phase 1: R-F = 0.07, and phase 3: R-F = 0.06).

Differences in the two phases became evident when considering the index case “adult only” and the resulting secondary infections. In total, *n* = 40 adults were identified as index cases in phase 1 and *n* = 32 in phase 3. These infections resulted in a total of 179 secondary infections (R-F = 4.48) in phase 1 and 15 secondary infections in phase 3 (R-F = 0.47, *p* = 0.002). Just as in the case of “child only” as index, more children (phase 1: R-F = 3.93 and phase 3: R-F = 0.34, *p* = 0.001) than adults (phase 1: R-F = 0.55 and phase 3: R-F = 0.13) became infected by positive adults.

With comparable numbers of affected schools and index cases, the number of subsequent cases decreases significantly from phase 1 to phase 3. This becomes particularly obvious when looking at the transmission from adults to children. An average of 2.44 cases was reported per infection event in phase 1, of these *n* = 1.19 were classified as index cases and *n* = 1.25 as secondary cases. In phase 3, an average of 1.57 cases occurred per infection event, of which 1.18 were index cases and 0.39 were secondary cases (*p* = 0.046).

### Infection Events in Pre-schools

An overview of the COVID-19 infection events at pre-schools subdivided into phases during the study period is given in Table 3.

**Table 3:** Infection incidence in pre-schools in Mecklenburg-Western Pomerania subdivided into phases subdivided by time phases depending on infection control measures in place, *N* = 438.

	**Phase 1** **(CW32-CW51, 2020)** ***n* = 84**	**Phase 2** **(CW52, 2020–CW7, 2021)** ***n* = 60**	**Phase 3** **(CW8–CW15, 2021)** ***n* = 201**	**Phase 4** **(CW16-CW19, 2021)** ***n* = 93**	***p*-value** **Phase 1 vs.** **Phase 3**
**Pre-schools open**	**Yes**	**No**	**Yes**	**No**	
**County, no. (%)**					**0.001**
Hansestadt Rostock	15 (17.9)	7 (11.7)	11 (5.5)	12 (12.9)	
Landkreis Rostock	10 (11.9)	13 (21.7)	30 (14.9)	20 (21.5)	
Ludwigslust-Parchim	19 (22.6)	7 (11.7)	28 (13.9)	12 (12.9)	
Mecklenburgische Seenplatte	2 (2.4)	3 (5.0)	21 (10.4)	12 (12.9)	
Nordwest-Mecklenburg	7 (8.3)	7 (11.7)	34 (16.9)	9 (9.7)	
Schwerin	3 (3.6)	0 (0)	16 (8.0)	5 (5.4)	
Vorpommern-Greifswald	15 (17.9)	18 (30.0)	40 (19.9)	15 (16.1)	
Vorpommern-Rügen	13 (15.5)	5 (8.3)	21 (10.4)	8 (8.6)	
**Average cases per infection event**					
Cases overall	1.63	3.20	3.03	1.65	**0.002**
Index cases	1.07	1.27	1.18	1.12	0.070
Secondary cases	0.56	1.93	1.85	0.53	**0.007**
Index cases children	0.48	0.62	0.60	0.73	0.180
Index cases adults	0.60	0.65	0.58	0.39	0.512
Secondary cases children	0.19	1.18	1.18	0.43	**<0.001**
Secondary cases adults	0.37	0.75	0.67	0.10	0.120
Index cases overall, absolute no.	90	76	237	104	
Secondary cases overall, absolute no.	47	116	372	49	
R-Factor (R-F^*^)	0.52	1.53	1.57	0.47	
**Overall cases per infection event, no. (%)**					**0.005**
One case overall	62 (73.8)	38 (63.3)	112 (55.7)	67 (72.0)	
2–10 cases overall	22 (26.2)	15 (25.0)	78 (38.8)	25 (26.9)	
>10 cases overall	0 (0)	7 (11.7)	11 (5.5)	1 (1.1)	
**Affected person as index case, no. (%)**					0.337
Child only	36 (42.9)	24 (40.0)	97 (48.3)	60 (64.5)	
Adult only	47 (56.0)	30 (50.0)	97 (48.3)	32 (34.4)	
Child + adult^**^	1 (1.2)	6 (10.0)	7 (3.5)	1 (1.1)	
Index case child only, no. (R-F^*^)	39	26	111	66	
Secondary cases overall	19 (0.49)	25 (0.96)	57 (0.51)	17 (0.26)	0.729
Secondary cases adults	11 (0.28)	8 (0.31)	17 (0.15)	4 (0.06)	0.378
Secondary cases children	8 (0.21)	17 (0.65)	40 (0.36)	13 (0.20)	0.586
Index case adult only, no. (R-F^*^)	48	32	107	35	
Secondary cases overall	28 (0.58)	37 (1.16)	301 (2.81)	28 (0.80)	**<0.001**
Secondary cases adults	20 (0.42)	23 (0.72)	114 (1.07)	3 (0.09)	**0.008**
Secondary cases children	8 (0.17)	14 (0.44)	187 (1.75)	25 (0.71)	**<0.001**
**Index case child+adult, no**.	3	18	19	3	
(child | adult)	(1 | 2)	(11 | 7)	(10 | 9)	(2 | 1)	
Secondary cases overall	0	54	14	4	
(child | adult)	(0 | 0)	(40 | 14)	(10 | 4)	(2 | 2)	
**Measure for containment, no. (%)**					**<0.001**
Contact person quarantine	9 (11.7)	28 (48.3)	91 (45.5)	42 (45.2)	
Cohort quarantine	45 (58.4)	16 (27.6)	79 (39.5)	46 (49.5)	
Closure of the entire facility	23 (29.9)	14 (24.1)	30 (15.0)	5 (5.4)	

* *R-F, R-Factor*.

** *This category was applied when a child and an adult were documented as index cases because it could not be differentiated which person was infected first, for example, because symptoms may have occurred at the same time or two outbreaks may have occurred simultaneously*.

*Values in bold are considered to be statistically significant (p-value < 0.05)*.

In phase 1, *n* = 84 infection events occurred overall in pre-schools, with *n* = 90 index cases leading to *n* = 47 secondary cases (R-F = 0.52). Whereas, in phase 3 *n* = 201 infection events occurred, whereby *n* = 237 index cases led to *n* = 372 secondary cases (R-F = 1.57). Comparing phase 1 to phase 3 (pre-schools were open), significantly more cases overall occurred in phase 3 with significantly more secondary cases (*p* = 0.002; *p* = 0.007). When comparing the two phases, there were no significant differences in the distribution between children and adults as index cases. In terms of secondary cases, significantly more children were affected in phase 3 compared to phase 1 (*p* < 0.001), whereas no differences were seen in adults (*p* = 0.120).

If the index case was a child, there were no differences in the number of secondary cases and the individuals affected between the two phases. In the first phase infections in children in pre-schools caused an average of 0.49 secondary cases and the corresponding number in the third phase was 0.51 secondary cases.

If the index case was an adult, there were significantly more secondary cases in the 3rd phase compared to the first phase (1st phase, R-F = 0.58; 3rd phase R-F = 2.81; *p* < 0.001), with more secondary cases among adults (*p* = 0.008), as well as among children (*p* < 0.001). Infections in adults in pre-schools caused an average of 0.42 secondary cases in adults and 0.17 secondary cases in children in the first phase, whereas infections in adults in the third phase caused 1.07 secondary cases in adults and 1.75 secondary cases in children. Accordingly, in the third phase the probability of transmission of adults as index case was five times higher compared to children.

### Impact of Hygiene Measures in Schools and Pre-schools on Extent of Outbreak Occurrence

To examine the impact of hygiene measures on the magnitude of outbreak occurrence, a linear regression model was set up with the number of total secondary cases per infection event as the dependent variable. The linear regression model is presented in Table 4. The wearing of face masks by children and adults was included in the model, as these hygiene measures differed between schools and pre-schools.

**Table 4:** Linear regression models with dependent variable number of secondary cases, *N* = 913.

**Variable**	**Univariate**		**Common multivariate model**		**Multivariate model adults**		**Multivariate model children**	
	***B* (95% CI)**	***p*-value**	***B* (95% CI)**	***p*-value**	***B* (95% CI)**	***p*-value**	***B* (95% CI)**	***p*-value**
Number of index cases overall	0.534 (0.143, 0.926)	**0.008**	0.092 (−0.315, 0.499)	0.657	0.088 (−0.317, 0.494)	0.669	0.145 (−0.262, 0.552)	0.484
**Affected person as index case**								
Child only (1) Adult only (2) Child + adult (3)^*^	1.519 (1.159, 1.878)	**<0.001**	1.340 (0.931, 1.748)	**<0.001**	1.343 (0.936, 1.750)	**<0.001**	1.303 (0.893, 1.712)	**<0.001**
**Face mask obligatory for adults**								
No (0) Yes, under certain circumstances (1) ^#^ Yes, while in the building (2)	−0.902 (−1.343, −0.461)	**<0.001**	−1.830 (−3.127, −0.532)	**0.006**	−1.941 (−2.886, −0.996)	**<0.001**		
**Face mask obligatory for children**								
No (0) No/Yes, under certain circumstances (1)^$^ Yes, under certain circumstances (2)^#^ Yes, while in the building (3)	−0.277 (−0.430, −0.125)	**<0.001**	−0.065 (−0.583, 0.453)	0.806			−0.565 (−0.944, −0.186)	**0.004**
**Setting**								
School (0) Pre-school (1)	0.592 (0.181, 1.003)	**0.005**	−1.372 (−2.406, −0.337)	**0.009**	−1.292 (−2.107, −0.476)	**0.002**	−1.275 (−2.311, −0.238)	**0.016**

*The coding of the respective categories of the nominal variables are shown in parentheses. For multivariate linear regression models time and location confounders were considered by CW (CW32, 2020 until KW19, 2021) and county of the school/pre-school and as dummy-coded variables included in the model*.

*Values in bold are considered to be statistically significant (p-value < 0.05)*.

* *This category was applied when a child and an adult were documented as index cases because it could not be differentiated which person was infected first, for example, because symptoms may have occurred at the same time or two outbreaks may have occurred simultaneously*.

# *“Yes, under certain circumstances” was hereby applied if the mask obligation existed not during the entire time spent in the school building/pre-school (Table 1). For schools, for example, this applies if there was no mask obligation during lessons and in pre-schools if there was no mask obligation for staff during pedagogical work with the children*.

$ *For schools with a primary and secondary part, different regulations applied with regard to the mask obligation for children of different age group*.

In univariate analysis, mandatory masking of adults (*B* = −0.9; *p* < 0.001) and mandatory masking of children (*B* = −0.3; *p* < 0.001) each resulted in a significant reduction in the number of secondary cases. An increasing number of index cases leads to an increase in secondary cases (*B* = 0.5; *p* = 0.008). Infections of adults and adults + children as index cases were positively associated with more secondary cases (*B* = 1.5; *p* < 0.001) (Table 4).

Due to an existing multicollinearity in the common multivariate regression model between the variables face mask obligation for children and face mask obligation for adults, two further separate regression models were set up (Table 4, Multivariate Model Adults and Multivariate Model Children). Requiring adults and children to wear masks significantly reduced the likelihood of secondary cases (Model Adults: *B* = −1.9; *p* < 0.001; Model Children: *B* = −0.6; *p* = 0.004) and having an adult or child+adult as index case increased the likelihood of secondary cases (*B* = 1.3; *p* < 0.001); Model Adults: *R*^2^ = 0.152, corrected *R*^2^ = 0.106; Model Children: *R*^2^ = 0.145, corrected *R*^2^ = 0.099. Cohens *f*^2^ = 0.12 (Model Adults) and Cohens *f*^2^ = 0.11 (Model Children) represent a small effect size of the multivariate linear regression models.

## Discussion

Due to the highly dynamic nature of the COVID-19 pandemic, especially during the winter season in 2020/2021, and due to the simultaneous start of COVID-19 vaccination, there were repeated adjustments of Corona hygiene regulations for schools and pre-schools in Mecklenburg-Western Pomerania, Germany. This prospective observational study aimed to map the dynamic changes in infection events at schools and pre-schools and to highlight factors influencing the extent of an outbreak while taking hygiene measures into account.

To date, several studies provide evidence that schools are not drivers of the pandemic, but may contribute to the reduction of the reproduction number, depending on other hygiene measures in the population (24–26). Consistent with others, our data from schools and pre-schools in Mecklenburg-Western Pomerania suggest that the risk of a COVID-19 outbreak in these institutions increases if the index case is an adult (26).

In schools, outbreaks with secondary cases occurred more frequently in the first phase, whereas outbreaks in pre-schools occurred more frequently in the third phase. A recently published study by Loenenbach et al. provides preliminary evidence that as SARS-CoV-2 variant B.1.1.7 has become more widespread; the susceptibility and infectivity of children and adolescents has increased (18). Similar data are shown in the REACT_r12 study in England (19) and in a study from Scotland (20). The delta variant (B.1.617.2) of SARS-CoV-2 is spreading rapidly worldwide, and as of the end of June 2021, is the dominant virus variant in Germany, accounting for 59% of the total in Germany (27). Initial studies indicate that children were more likely to be infected with the delta variant of the virus than adults (19, 20). An increased risk of severe disease progression was not observed. As with the previous variants, the risk of severe disease progression in children and adolescents without previous illness is very low.

We could observe a comparable trend in pre-schools, but not in schools. However, an increase in infection cases in elementary schools in phase 3 suggests a shift of infections toward the age group <12. It should be emphasized that there were comprehensive adaptations of hygiene concepts in schools e.g., incidence-dependent alternating lessons, as well as an obligation to wear face masks during lessons in the third phase—possibly reducing outbreak events. On the other hand, voluntary point-of-care (PoC) testing has been implemented in schools since 03/21, increasing the likelihood of detecting asymptomatic cases. Mandatory PoC testing at schools did not begin until the end of April 2021, CW17. No mandatory PoC testing was conducted in pre-schools. In our study, we did not see an increase in the number of cases in schools since March 2021. In both schools and pre-schools, symptomatic children and adults had to stay at home. In Mecklenburg-Western Pomerania, both teachers and pedagogical staff were offered vaccinations from the beginning of March 2021. However, the acceptance rate of the offers cannot be verified as there is no consistent vaccination surveillance in Germany.

Interestingly, the strongest predictor of the extent of the outbreak in our study was found to be the teacher/caregiver mask obligation. Furthermore, requiring children to wear masks may also reduce the number of secondary cases. The existing multicollinearity between the variables face mask obligation for children and face mask obligation for adults in the multivariate regression model leads to the fact that not both variables should be considered in one regression model, and even after separation into two regression models, the estimates should be considered with caution since estimates for children may be at least partially explained by adult mask-wearing and vice-versa. Nevertheless, since adults were more often the index case, adult mask-wearing is particularly effective. Of course, children wearing face masks reduces the risk of infection and consequently the number of secondary cases, but mask-wearing is not recommended in young children under 6 years of age, as they cannot use it properly.

When comparing the hygiene measures between schools and pre-schools, an important difference emerges. In pre-schools, masks were not mandatory at any time during the educational work, i.e., the interaction between staff and child. Other hygiene measures were largely comparable between schools and pre-schools. The implementation of hygiene measures following the regulations can only be assumed here. As part of another project, we carried out school inspections, whereby we monitor infection events at schools and the compliance to hygiene regulations. In these inspections we have so far not been able to detect any gross violations (unpublished data). An analysis by Philipps et al. showed that outbreak size in pre-schools and primary schools may be reduced by smaller group sizes and grouping of siblings (28).

It is important to highlight that the temporal subdivision of the phases is simplified and the containment measures were heterogeneous and complex, depending on the incidence of each county, especially in the 3rd phase (school and pre-school opened). Therefore, time and location were included as potential confounders in our model. Another important aspect is, that further to the different age distribution of children in schools and pre-schools, the staff-child interactions are not comparable between these two settings. Therefore, univariate analyses of the infection events were performed separately and in the multivariate regression model the setting (school/pre-school) was considered.

Nevertheless, the presented data on the incidence of infections in pre-schools in the third phase is of concern and should receive more focus in the public debate—especially in light of the fact that new virus mutations may lead to altered transmission and infectiousness in children and adolescents.

In our opinion, a mask requirement for caregivers in pre-schools should be considered. For example, instead of medical masks, colorful fabric masks could be recommended to make children feel more comfortable. The educational work could be impaired by such a measure, yet mouth-nose covering is one effective preventive method to contain the COVID-19 pandemic (29, 30). Given that educational professionals and teaching staff in Germany have been prioritized for vaccination, a reduction in the incidence of infection in schools and pre-schools can be assumed. However, verification of vaccination status is not permissible, so that a mask requirement should be reconsidered.

Limitations of this study are especially the insufficient information on SARS-CoV-2 positive tested persons at schools and pre-schools, such as demographics, medical history, number of contact persons and symptoms since illness onset to further specify the factors influencing the extent of the outbreaks. Furthermore, it must be emphasized that contact persons of infected persons have not been tested consistently, but have often been quarantined based on contact only, so that the number of SARS-CoV-2 positive cases may be underestimated. In particular, children and adolescents are often asymptomatic, so that cases may go undetected. As mentioned above, the temporal breakdown into phases is simplified and based on the guidelines of the state of Mecklenburg-Western Pomerania, Germany. Furthermore, during phase 1 the hygiene measures were partly different at one school, for example, primary school students did not have to wear a mask, whereas secondary school students had to wear a mask e.g., in the corridors and during breaks. This was therefore considered in the multivariate analysis.

With the start of the new school year 2021/2022, children and adolescents will be among the age groups with the lowest vaccination coverage for COVID-19. Therefore, in the absence of strict adherence to hygiene measures, a concentrated spread of COVID-19, including outbreaks, might be expected in these age groups.

In principle, however, it can be assumed that the established hygiene rules (distance, frequent handwashing, wearing a mask) will also protect against new variants of SARS-CoV-2. Due to the possible increased susceptibility of children, these measures must be implemented even more consistently, especially in the age group <12 years. A focus should therefore be placed on effective protection concepts in pre-school and elementary school settings and in after-school care centers.

In conclusion, the dynamics of infection events differ between schools and pre-schools over time. Considering the respective applicable hygiene measures in schools and pre-schools in the study region, as well as temporal and spatial factors, the present study indicates that outbreak events at pre-schools and schools are particularly potentiated when an adult is the index case. Thus, an obligation for adults to wear face masks might be an important measure to contain outbreaks, particularly in pre-schools during educational work.

## Data Availability Statement

The raw data supporting the conclusions of this article will be made available by the authors, without undue reservation.

## Author Contributions

MS, PL, WH, ER, and AK: conceptualization. MS, PL, ME, and AK: methodology. MS, ME, PL, WH, and AK: investigation. SB and ML: resources. MS and AK: writing—original draft preparation. MS, PL, ME, WH, and ER: writing—review and editing. MS, WH, AK, and ER: funding acquisition. All authors have read and agreed to the published version of the manuscript.

## Funding

This study was financially supported by the Ministry of Economic Affairs, Labor and Health Mecklenburg-Western Pomerania.

## Conflict of Interest

The authors declare that the research was conducted in the absence of any commercial or financial relationships that could be construed as a potential conflict of interest.

## Publisher's Note

All claims expressed in this article are solely those of the authors and do not necessarily represent those of their affiliated organizations, or those of the publisher, the editors and the reviewers. Any product that may be evaluated in this article, or claim that may be made by its manufacturer, is not guaranteed or endorsed by the publisher.
